# Injury Severity Score Precision for Determining Undertriage in Trauma Consultation Patients: A Retrospective Study

**DOI:** 10.7759/cureus.73341

**Published:** 2024-11-09

**Authors:** C. Michael Dunham, Gregory S Huang, Elisha A Chance, Barbara M Hileman

**Affiliations:** 1 Trauma, Critical Care, and General Surgery, Mercy Health, St. Elizabeth Youngstown Hospital, Youngstown, USA; 2 Trauma and Neuroscience Research, Mercy Health, St. Elizabeth Youngstown Hospital, Youngstown, USA

**Keywords:** adverse outcomes, consult patients, glasgow coma scale, injury severity score, intracranial hemorrhage, precision, preexisting medical conditions, trauma centers, trauma mortality, undertriage

## Abstract

Objectives

Because the Injury Severity Score (ISS) has not been fully endorsed for determining trauma undertriage, we investigated its precision. This study aimed to 1) compute undertriage proportions using the Peng and Cribari methods; 2) compare risk conditions and outcomes for patients with ISS ≥16 who were classified according to an activation or consultation status; 3) calculate proportions of patients with ISS ≥16, without and with qualifications (death or increased length of stay), as potential categories for assessing undertriage in trauma consultation patients; and 4) compute the undertriage proportion among trauma consultation patients by using an intracranial hemorrhage (ICH)-Glasgow Coma Scale score (GCS)-ISS categorization method and employing targeted electronic medical record audits (EMRA).

Methodology

Age, ISS, GCS, activation status (full, partial, or consultation), injury mechanism, death, intensive care stay, and hospital stay were obtained from the trauma registry. An adverse outcome (AO) was death, intensive care stay ≥two days, or hospital stay >five days. ICH status, clinical details, and comorbidity were obtained from EMRA. Each consultation patient was assigned to an ICH-GCS-ISS category with targeted EMRA.

Results

Of 2,076 consecutive trauma center admissions, 405 had full activation, 640 had partial activation, and 1,031 had consultation. Using Peng and Cribari methods, undertriage proportions were 64.2% and 21.7%, respectively. Compared with consultation patients with ISS ≥16, full or partial activation patients with ISS ≥16 had much higher proportions (p<0.0001) of non-fall mechanisms, age <70 years, GCS <15, ISS ≥25, mortality, intensive care admission, and hospital stay >five days. The proportions of consultation nonqualified ISS ≥16 undertriage and ISS ≥16-AO undertriage were 21.1% (218 ISS ≥16÷1,031) and 9.8% (101 ISS ≥16-AO÷1,031), respectively. Of the 101 ISS ≥16-AO patients, 79.2% were aged ≥70 years or had comorbidity. The consultation ISS ≥16-death undertriage proportion was 1.5% (15 ISS ≥16-deaths÷1,031). The ICH-GCS-ISS consultation categorization undertriage proportion was 11.0% (113÷1,031). ICH-GCS-ISS undertriage proportions were as follows: ICH with GCS <15, 77/77; no ICH with ISS ≥16 (EMRA for major injury), 14/60; scene ICH & GCS 15 & ISS ≥16, 0/43; transfer ICH & GCS 15 & ISS ≥16 (EMRA for intracranial mass effect), 22/69; no ICH with ISS <16, 0/629; and ICH & GCS 15 & ISS <16, 0/153.

Conclusions

Because ISS ≥16 consultation and activation patient risk conditions and outcomes are dissimilar, commingling these cohorts for undertriage assessment is dubious. Death and increased length of stay-qualified ISS ≥16 with EMRA can be useful to assess undertriage in trauma consultation patients. Because most ISS ≥16-AO consultations were elderly or had comorbidity, the benefit of trauma activation is uncertain. Consultation ICH-GCS-ISS categorization with EMRA was needed in only 12%, indicating a relatively facile undertriage method.

## Introduction

A recent search in the National Library of Medicine produced over 850 publications regarding the topic of emergency department triage. The first publication was cited in 1964 [1] and continues through the current year [2,3]. In total, 480 citations dealt with trauma triage, with most of these addressing the issue of undertriage. A trauma undertriage patient is typically described as an individual with a major injury (Injury Severity Score (ISS) ≥16 or need for trauma intervention) who did not undergo a full trauma team activation [4-11].

A common method for computing a trauma center undertriage proportion is the use of a matrix where the undertriage proportion is the number of patients with ISS ≥16 and without full trauma team activation (partial activation plus no activation) divided by the total number of patients with ISS ≥16 (full trauma team activation, plus partial activation, plus no activation) [12]. We found several studies where the aforementioned undertriage matrix data were presented in the manuscript [4-7,9-11]. According to these seven publications, the undertriage proportion range was 15.3-84.6% and had a random effects total (meta-analysis) proportion of 52.2% (heterogeneity p<0.0001). Although the American College of Surgeons Committee on Trauma considers that an undertriage rate of <5% is acceptable [12], none of these studies fulfilled that goal. The wide undertriage proportion rand and high meta-analysis proportion suggest that 1) this undertriage method may be affected by variations in triage criteria among the trauma systems analyzed; 2) emergency department staff perceptions of a need for trauma team activation may fluctuate; 3) ISS ≥16 may be an imprecise metric to connote major injury in non-trauma team patients; 4) the methodological components are incoherent; or 5) systematic undertriage assessment is an otherwise complex issue.

During the past four years, several investigations have qualified or replaced ISS ≥16 as a designation for major injury by delineating those who had a need for trauma intervention [4,5,13-17]. Some of these researchers have specifically qualified ISS ≥16 with a need for trauma intervention to designate those with a major injury in cohorts of consultation (nonactivation) patients [5,15-17]. That is, the proportion of consult patients with ISS ≥16 and a need for a trauma intervention were considered to represent trauma center undertriage. Of these four consultation investigations, the nonqualified ISS ≥16 proportions ranged from 9.1-17.2% [5,15-17].

Of relevance to the current study, numerous trauma investigations have shown that hospital mortality is associated with the severity of anatomic injury (using the ISS) [18-21], increased age [22-25], preexisting medical conditions (PEMC) [26-29], decreased Glasgow Coma Scale score (GCS) [30-35], and intracranial hemorrhage (ICH) [31,34,36-39]. Virtually all of these studies included trauma patient cohorts that were either a combination of activation and nonactivation patients or only activation patients [40]. A recent publication focusing solely on trauma consultation patients showed that death and increased length of stay had associations with ISS ≥16, age ≥70, PEMC, decreased GCS, and ICH [40].

The current study has multiple objectives. The first aim was to compute undertriage proportions using the Peng and Cribari methods [12]. The second objective was to compare risk conditions and outcomes for patients with ISS ≥16 who were classified according to an activation or consultation status. The third aim was to compute proportions of ISS ≥16, without and with qualifications, as potential categories for assessing undertriage in trauma consultation patients. The final objective was to compute an undertriage proportion in trauma consultation patients using an ICH-GCS-ISS categorization method and employing targeted electronic medical record (EMR) audits.

## Materials and methods

Ethics statements

The current retrospective study was reviewed by the local institutional review board (IRB) and determined to be qualified for exemption according to Exempt Category 4 (Bon Secours Mercy Health, IRB number: 2024-Trauma-Dunham). The need for informed consent was waived.

Study design and population

The parent group data source was adult patients admitted to a level I trauma center in Northeast Ohio from January 21 to July 21 during each year 2018, 2019, and 2020, as described in a previous publication [41]. Of the total 2,076 trauma patients, 405 had full trauma team activation, 640 had partial trauma team activation, and 1,031 had trauma consultation (nonactivation).

Inclusion and exclusion criteria

In the current investigation, a consecutive subset of trauma consultation (non-activation) patients and a subset of activation patients (full or partial trauma team activation) aged ≥18 years were included. Patients aged <18 years or those with no trauma center admission were excluded.

Data collection

Some data were obtained from the trauma registry, and other information was obtained from the electronic medical records (EMR). Data from the trauma registry included mechanism of injury, age, GCS, ISS, blood pressure, hospital transfer or scene designation, hospital death, intensive care unit (ICU) stay (days), hospital stay (days), and activation or consultation status. Activation patients were those with full trauma team activation or partial trauma team activation, whereas consultation patients received no activation process. Information from the EMR included consultation patient presence or absence of ICH, preexisting medical conditions (PEMC), and hospital discharge to hospice. These three inquiries were pursued sequentially in each trauma consultation patient to determine the study results.

The presence or absence of ICH in consultation patients was determined by a method described previously [36]. The list of preexisting medical diseases considered in the computation of a PEMC score for consultation patients emanated from the Charlson Comorbidity Index [42]. The details of computing the PEMC score have been provided in an earlier study [40]. The PEMC score results were 0 (no PEMC), 1 (1 PEMC), or 2 (≥2 PEMC).

To determine hospice discharge for trauma consultation patients, palliative care was typed into the EMR search engine panel and submitted. Any palliative care consultation(s) performed during the trauma center admission were reviewed to determine if the patient was discharged to hospice. Deaths were the sum of the hospital deaths and patients discharged to hospice. Other investigators of trauma patients with ICH have considered discharge to hospice to be a fatal, terminal event [43]. When deaths were compared between consultation and activation patients, only the in-hospital, non-hospice deaths were considered. Adverse outcomes (death or increased length of stay) were death, intensive care unit (ICU) stay ≥two days, or hospital stay >five days. Other researchers consider that the need for intensive care is an indication of undertriage in non-activation trauma patients [44].

Activation and consultation ISS ≥16 undertriage methods

As a reference, the Peng method for describing trauma center undertriage was computed as the number of patients with ISS ≥16 but without full trauma team activation (partial activation plus consultation patients) divided by the total number of patients with ISS ≥16 (full trauma team activation, plus partial trauma team activation, plus trauma consultation) [12]. We modified the Peng method by computing the undertriage proportion as the number of consultation patients with ISS ≥16 divided by the total number of patients with ISS ≥16 (activation plus consultation patients). This procedure has precedence in a peer-reviewed study [8]. The Cribari undertriage method was calculated as the number of patients with ISS ≥16 but without full trauma team activation (partial activation plus consultation patients) divided by the total group of patients who had partial activation or consultation [12].

Consultation patient ISS ≥16 undertriage methods

We also modified the Cribari method by computing the proportion of consultation patients with ISS ≥16 divided by the total group that had undergone consultation. A similar approach with a focus solely on consultation patients and the proportion of patients with ISS ≥16 has precedence in the peer-reviewed literature [15-17]. Qualifications of consultation patients with ISS ≥16 were made by delineating cohorts with death and adverse outcomes (death or increased lengths of stay). These ISS ≥16 qualifications were used to delineate cohorts of consultation patients that were likely to represent undertriage. Other studies have qualified consultation patients with ISS ≥16 and the need for trauma interventions [15-17].

Consultation patient ICH-GCS-ISS categorization undertriage method

Another systematic audit was undertaken to delineate consultation patients who might have had undertriage. The consultation patients were classified according to five mutually exclusive categories based on ICH, GCS and ISS characteristics: 1) ICH & GCS <15; 2) No ICH & ISS ≥16; 3) ICH & GCS 15 & ISS ≥16; 4) No ICH & ISS <16; and 5) ICH & GCS 15 & ISS <16. Cases of inappropriate consultation triage were assigned to group 1) ICH & GCS <15, whereas cases of appropriate consultation triage were assigned to groups 4) No ICH & ISS <16 and 5) ICH & GCS 15 & ISS <16. EMR audits were performed on each patient in groups 2) No ICH & ISS ≥16 and 3) ICH & GCS 15 & ISS ≥16 to determine whether consultation triage was appropriate or inappropriate.

Statistical analysis

Continuous data are presented as the mean ± standard deviation, whereas categorical variables are reported as frequency count and percentage. T-tests were performed to test differences between two groups of continuous data. For dichotomous proportional data conforming to a 2 x 2 contingency table format, the two-tailed Fisher exact test was employed. The odds ratio (OR) and risk ratio (RR) were computed to quantify proportional differences. For proportional data conforming to a 2 x 5 contingency table format, the Chi-square test was used. Additionally, multivariate logistic regression analysis was used to assess independent variable associations relative to dichotomous dependent variables.

The proportions of those with a fall mechanism, age ≥70 years, GCS <15, ISS ≥25, systolic blood pressure <100 millimeters of mercury (mmHg), hospital mortality, adverse outcomes, ICU admission, and hospital stay >five days were compared between ISS ≥16 patients without full trauma team activation and ISS ≥16 patients with full trauma team activation (two-tailed Fisher exact test). Moreover, the proportions of those with a fall mechanism, age ≥70 years, GCS <15, ISS ≥25, systolic blood pressure <100 mmHg, hospital mortality, adverse outcomes, ICU admission, and hospital stay >five days were compared between ISS ≥16 patients undergoing trauma consultation and ISS ≥16 patients with trauma activation (full or partial trauma team activation) (two-tailed Fisher exact test). Stepwise multivariate regression analysis was used to delineate whether risk conditions (fall mechanism, age ≥70 years, GCS <15, ISS ≥25, and systolic blood pressure <100 mmHg) had an independent association in ISS ≥16 patients undergoing trauma activation (full or partial trauma team activation) when compared to ISS ≥16 patients undergoing trauma consultation.

For activation patients, the hospital mortality proportion was compared between those without and with ISS ≥16 (two-tailed Fisher exact test). For consultation patients, the hospital mortality proportion was compared between those without and with ISS ≥16 (two-tailed Fisher exact test). Stepwise multivariate regression analysis was used to delineate whether risk conditions (fall mechanism, age ≥70 years, GCS <15, ISS ≥25, and systolic blood pressure <100 mmHg) and outcomes (hospital mortality, adverse outcomes, ICU admission, and hospital stay >five days) had an independent association in all patients undergoing trauma activation (full or partial trauma team activation) compared to all patients undergoing trauma consultation.

Proportions of those with a fall mechanism, age ≥70 years, GCS <15, ISS ≥25, systolic blood pressure <100 mmHg, hospital mortality, adverse outcomes, ICU admission, and hospital stay >five days were also compared between ISS ≥16 patients undergoing consultation and ISS ≥16 patients receiving full trauma team activation (two-tailed Fisher exact test). Stepwise multivariate regression analysis was used to delineate whether risk conditions (fall mechanism, age ≥70 years, GCS <15, ISS ≥25, and systolic blood pressure <100 mmHg) had an independent association in ISS ≥16 patients undergoing full trauma team activation compared to ISS ≥16 patients receiving trauma consultation. Additionally, the proportions of those with a fall mechanism, age ≥70 years, GCS <15, ISS ≥25, systolic blood pressure <100 mmHg, hospital mortality, adverse outcomes, ICU admission, and hospital stay >five days were compared between ISS ≥16 patients undergoing consultation and ISS ≥16 patients receiving partial trauma team activation (two-tailed Fisher exact test). Stepwise multivariate regression analysis was used to delineate whether risk conditions (fall mechanism, age ≥70 years, GCS <15, ISS ≥25, and systolic blood pressure <100 mmHg) and outcomes (hospital mortality, adverse outcomes, ICU admission, and hospital stay >five days) had an independent association in ISS ≥16 patients undergoing partial trauma team activation compared to ISS ≥16 patients undergoing trauma consultation.

The trauma consultation nonqualified ISS ≥16 proportion was compared to the trauma consultation ISS ≥16 with adverse outcome proportion (two-tailed Fisher exact test). Further, the trauma consultation nonqualified ISS ≥16 proportion was compared to the trauma consultation ISS ≥16 with death proportion (two-tailed Fisher exact test).

The mortality proportions for the five ICH-GCS-ISS consultation categories were compared (Chi-square test). The adverse outcome proportions for the five ICH-GCS-ISS consultation categories were compared (Chi-square test). The mortality proportions for ICH-GCS-ISS consultation category groups 1-3 were compared to those for groups 4 and 5 (two-tailed Fisher exact test). The adverse outcome proportions for ICH-GCS-ISS consultation category groups 1-3 were compared to those for groups 4 and 5 (two-tailed Fisher exact test). The undertriage proportions for the ICH-GCS-ISS consultation categorical method were compared to the undertriage proportion computed as the nonqualified ISS ≥16 patients among all consultation patients (two-tailed Fisher exact test).

Data were entered into Excel 2010 (Microsoft Corp., Redmond, WA, USA) and imported into SAS System for Windows (release 9.2, SAS Institute Inc., Cary, NC, USA). The significance level for the p-value was set at <0.05.

## Results

The parent group consisted of 2,076 consecutive trauma center admissions studied over an 18-month period (January 21 to July 21 during each year 2018, 2019, and 2020) [41]. The current study focused on 1,031 trauma consultation, non-activation patients. The patient characteristics are presented in Table 1. Most patients had a fall mechanism, one-half were age ≥70 years, one-half had a PEMC, and proportions of ISS ≥16 and intracranial hemorrhage were substantial.

**Table 1:** Characteristics of the trauma consultation patients Data are presented as mean ± standard deviation or n (percentage). PEMC: preexisting medical condition.

Variable or Group	Patients (N=1,031)
Fall	774 (75.1%)
Motorized crash	154 (14.9%)
Penetrating injury	7 (0.7%)
Other	96 (9.3%)
Injury Severity Score ≥16	218 (21.1%)
Glasgow Coma Scale score <15	143 (13.9%)
Intracranial hemorrhage	342 (33.2%)
Age (years)	65.4 ± 21
Age ≥70 years	508 (49.3%)
PEMC score 0	498 (48.3%)
PEMC score 1	241 (23.4%)
PEMC score 2	292 (28.3%)
PEMC score 1 or 2	533 (51.7%)
Hospital transfer	630 (61.1%)
Hospital death	25 (2.4%)
Hospital death plus hospice discharge	28 (2.7%)
Intensive care unit	156 (15.1%)
Hospital stay (days)	3.9 ± 3.4
Hospital stay >5 days	210 (20.4%)

Activation and consultation ISS ≥16 undertriage methods

For comparison, the Peng undertriage proportion was 64.2% (363 patients with ISS ≥16 but without full trauma team activation (consultation plus partial trauma team activation patients) ÷ all 565 patients with ISS ≥16. The numbers of consultation patients with ISS ≥16 (without activation) and activation patients with ISS ≥16 were 218 and 347, respectively. The Peng modification undertriage proportion was 38.6% (218 ÷ 218 plus 347).

The comparison of patients with ISS ≥16 who did or did not undergo full trauma team activation is presented in Table 2. The patients with ISS ≥16 and full trauma team activation had substantially greater proportions of non-fall mechanisms, age <70 years, GCS <15, ISS ≥25, systolic blood pressure <100 mmHg, hospital mortality, adverse outcomes, ICU admission, and total stay >five days than the other patients with ISS ≥16.

**Table 2:** Comparison of patients with ISS ≥16 who did or did not undergo full trauma team activation Adverse outcomes included hospital death, ICU stay ≥two days, or hospital stay >five days ISS: Injury Severity Score; χ2: Chi-square value generated using the two-tailed Fisher exact test; OR: Odds ratio; RR: Risk ratio; GCS: Glasgow Coma Scale score; SBP: Systolic blood pressure; mmHg: Millimeters of mercury; ICU: Intensive care unit.

Full Trauma Team Activation	Yes	No	p-value	χ^2^	OR	RR
Number	202 (35.8%)	363 (64.2%)	–	–	–	–
Non-fall mechanism	153 (75.7%)	128 (35.3%)	<0.0001	85.1	5.7	2.2
Age <70 years	163 (80.7%)	194 (53.4%)	<0.0001	41.4	3.6	1.5
GCS <15	140 (69.3%)	99 (27.3%)	<0.0001	94.0	6.0	2.5
ISS ≥25	124 (61.4%)	79 (21.8%)	<0.0001	88.5	5.7	2.8
SBP <100 mmHg	61 (30.2%)	14 (3.9%)	<0.0001	78.2	10.8	7.8
Hospital mortality	50 (24.8%)	20 (5.5%)	<0.0001	44.3	5.6	4.5
Adverse outcomes	186 (92.1%)	188 (51.8%)	<0.0001	94.1	12.1	1.9
ICU admission	168 (83.2%)	153 (42.2%)	<0.0001	89.0	6.8	2.0
Total stay >5 days	130 (64.4%)	152 (41.9%)	<0.0001	26.2	2.5	1.5

The comparison of activation (full or partial trauma team) and consultation patients with ISS ≥16 is presented in Table 3. The differences between the two groups were comparable to those presented in Table 2. Of the 92 fall patients with activation and ISS ≥16, 78 (84.8%) fell down stairs or from a standing height. Of the 192 fall patients with consultation and ISS ≥16, 177 (92.2%) fell down stairs or from a standing height.

**Table 3:** Comparisons of activation and consultation patients with ISS ≥16 Adverse outcomes include hospital death, ICU stay ≥two days, or hospital stay >five days ISS: Injury Severity Score; χ2: Chi-square value generated using two-tailed Fisher exact test; OR: Odds ratio; RR: Risk ratio; GCS: Glasgow Coma Scale score; SBP: Systolic blood pressure; mmHg: Millimeters of mercury; ICU: Intensive care unit.

Variable	Activations	Consultations	p-value	χ^2^	OR	RR
Number	347 (61.4%)	218 (38.6%)	–	–	–	–
Non-fall mechanism	255 (73.5%)	26 (11.9%)	<0.0001	203.0	20.5	6.2
Age <70 years	276 (79.5%)	81 (37.2%)	<0.0001	103.4	6.6	2.1
GCS <15	189 (54.5%)	50 (22.9%)	<0.0001	54.4	4.0	2.4
ISS ≥25	155 (44.7%)	48 (22.0%)	<0.0001	29.8	2.9	2.0
SBP <100 mmHg	67 (19.3%)	8 (3.7%)	<0.0001	28.4	6.3	5.3
Hospital mortality	58 (16.7%)	12 (5.5%)	<0.0001	15.5	3.5	3.0
Adverse outcomes	275 (79.3%)	99 (45.4%)	<0.0001	68.5	4.6	1.8
ICU admission	246 (70.9%)	75 (34.4%)	<0.0001	72.7	4.6	2.1
Total stay >5 days	204 (58.8%)	78 (35.8%)	<0.0001	28.4	2.6	1.6

Multivariate logistic regression analysis of all activation and consultation patients with ISS ≥16 (n=565) demonstrated that activation had independent associations with non-fall mechanisms (p<0.0001), age <70 years (p<0.0001), GCS <15 (p<0.0001), ISS ≥25 (p=0.0081), and systolic blood pressure <100 mmHg (p=0.0052). The r-square value was 0.50. For activation patients, the hospital mortality proportion was greater with ISS ≥16 (16.7% (58/347)) than with ISS <16 (0.9% (6/698); p<0.0001; OR=23.2; RR=19.4). For consultation patients, the hospital mortality proportion was greater with ISS ≥16 (5.5% (12/218)) than with ISS <16 (1.6% (13/813); p=0.0009; OR=3.6; RR=3.4). Multivariate logistic regression analysis of all activation and consultation trauma patients (n=2,076) showed that activation patients had independent associations with non-fall mechanisms (p<0.0001), age <70 years (p<0.0001), GCS <15 (p<0.0001), ISS ≥25 (p=0.0173), systolic blood pressure <100 mmHg (p=0.0027), and intensive care admission (p<0.0001). The r-square value was 0.40.

The comparison of full trauma team activation and consultation patients with ISS ≥16 is presented in Table 4. The differences between the two groups were comparable to those shown in Table 2. Multivariate logistic regression analysis of all full trauma team activation and consultation patients with ISS ≥16 (n=420) demonstrated that full trauma team activation had independent associations with non-fall mechanisms (p<0.0001), age <70 years (p=0.0010), GCS <15 (p<0.0001), ISS ≥25 (p=0.0001), and systolic blood pressure <100 mmHg (p<0.0001). The r-square value was 0.60.

**Table 4:** Comparisons of full trauma team activation and consultation patients with ISS ≥16 Adverse outcomes include hospital death, ICU stay ≥two days, or hospital stay >five days ISS: Injury Severity Score; FTTA: full trauma team activation; χ2: Chi-square value generated using the two-tailed Fisher exact test; OR: Odds ratio; RR: Risk ratio; GCS: Glasgow Coma Scale score; SBP: Systolic blood pressure; mmHg: Millimeters of mercury; ICU: Intensive care unit.

Variable	FTTA	Consultations	p-value	χ^2^	OR	RR
Number	202 (48.1%)	218 (51.9%)	–	–	–	–
Non-fall mechanism	153 (75.7%)	26 (11.9%)	<0.0001	174.6	23.1	6.4
Age <70 years	163 (80.7%)	81 (37.2%)	<0.0001	81.6	7.1	2.2
GCS <15	140 (69.3%)	50 (22.9%)	<0.0001	91.0	7.6	3.0
ISS ≥25	124 (61.4%)	48 (22.0%)	<0.0001	67.2	5.6	2.8
SBP <100 mmHg	61 (30.2%)	8 (3.7%)	<0.0001	53.7	11.4	8.2
Hospital mortality	50 (24.8%)	12 (5.5%)	<0.0001	30.9	5.7	4.5
Adverse outcomes	186 (92.1%)	99 (45.4%)	<0.0001	104.7	14.0	2.0
ICU admission	168 (83.2%)	75 (34.4%)	<0.0001	102.3	9.4	2.4
Total stay >5 days	130 (64.4%)	78 (35.8%)	<0.0001	34.3	3.2	1.8

The Cribari undertriage proportion was 21.7% (363 consultation or partial trauma team activation patients with ISS ≥16 ÷ 1,671 total consultation plus partial trauma team activation patients). The comparison of patients with ISS ≥16 who had a consultation or partial trauma team activation is presented in Table 5. The patients with ISS ≥16 and partial trauma team activation had significantly greater proportions of non-fall mechanisms, age <70 years, GCS <15, adverse outcomes, ICU admission, and total stay >five days than the consultation patients with ISS ≥16. Multivariate logistic regression analysis of all partial trauma team activation and consultation patients with ISS ≥16 (n=363) showed that partial trauma team activation had independent associations with non-fall mechanisms (p<0.0001), age <70 years (p=0.0126), GCS <15 (p=0.0031), and ICU admission (p=0.0041). The r-square value was 0.40.

**Table 5:** Comparisons of partial activation and consultation patients with ISS ≥16 Adverse outcomes include hospital death, ICU stay ≥two days, or hospital stay >five days ISS: Injury Severity Score; χ2: Chi-square value generated using two-tailed Fisher exact test; OR: Odds ratio; RR: Risk ratio; GCS: Glasgow Coma Scale score; SBP: Systolic blood pressure; mmHg: Millimeters of mercury; ICU: Intensive care unit.

Variable	Partial Activations	Consultations	p-value	χ^2^	OR	RR
Number	145 (39.9%)	218 (60.1%)	–	–	–	–
Non-fall mechanism	102 (70.3%)	26 (11.9%)	<0.0001	130.2	17.5	5.9
Age <70 years	113 (77.9%)	81 (37.2%)	<0.0001	58.2	6.0	2.1
GCS <15	49 (33.8%)	50 (22.9%)	0.0229	5.2	1.7	1.5
ISS ≥25	31 (21.4%)	48 (22.0%)	0.8851	0.1	–	–
SBP <100 mmHg	6 (4.1%)	8 (3.7%)	0.8205	0.1	–	–
Hospital mortality	8 (5.5%)	12 (5.5%)	0.9959	0.0	–	–
Adverse outcomes	89 (61.4%)	99 (45.4%)	0.0029	8.9	1.9	1.4
ICU admission	78 (53.8%)	75 (34.4%)	0.0002	13.4	2.2	1.6
Total stay >5 days	74 (51.0%)	78 (35.8%)	0.0039	8.3	1.9	1.4

Consultation patient ISS ≥16 undertriage methods

The nonqualified ISS ≥16 consultation patient frequency count was 218. Using this count, the modified Cribari consultation undertriage proportion was 21.1% (218 consultation patients with ISS ≥16 ÷ 1,031 total consultation patients). Among the patients with consultation, the ISS ≥16 with adverse outcomes frequency count was 101. Using this count, the ISS ≥16-adverse outcomes consultation undertriage proportion was 9.8% (101 consultation patients with ISS ≥16 and an adverse outcome ÷ 1,031 total consultation patients). Among the 101 ISS ≥16-adverse outcome consultation patients, the proportion with either age ≥70 years or PEMC present was 79.2% (80/101). Among the patients with consultation, the ISS ≥16 with adverse outcomes and age <70 years and no PEMC frequency count was 21. Using this count, the ISS ≥16-adverse outcomes with age <70 and no PEMC consultation undertriage proportion was 2.0% (21 consultation patients with ISS ≥16 and an adverse outcome with age <70 years and no PEMC ÷ 1,031 total consultation patients). Among the patients with consultation, the ISS ≥16 with death frequency count was 15. Using this count, the ISS ≥16-death consultation undertriage proportion was 1.5% (15 consultation patients with ISS ≥16 and death ÷ 1,031 total consultation patients). Among the 15 ISS ≥16-death consultation patients, the proportion with either age ≥70 years or PEMC present was 93.3% (14/15). The consultation patient undertriage proportion was greater in patients with nonqualified ISS ≥16 (21.1% (218/1,031)) than in those with ISS ≥16-adverse outcomes (9.8% (101/1,031); p<0.0001; OR=2.5; RR=2.2) and in those with ISS ≥16-deaths (1.5% (15/1,031); p<0.0001; OR=18.2; RR=14.5).

Consultation patient ICH-GCS-ISS categorization undertriage method

The consultation patients were classified according to five mutually exclusive categories based on ICH, GCS, and ISS characteristics. The five categories and their distribution and death and adverse outcome proportions are displayed in Table 6. Groups 1-3 had substantially greater death and adverse outcome proportions than groups 4 and 5. Similarly, consultation patients with ISS ≥16 had considerably greater death and adverse outcome proportions than those with ISS <16. The ISS ≥16 proportion for group 1 (ICH & GCS <15) was 59.7% (46/77), and for groups 1-3 was 87.6% (218/249).

**Table 6:** Trauma consultation patient ICH-GCS-ISS categorical results Adverse outcomes include hospital death, intensive care unit stay ≥two days, or hospital stay >five days ICH: Intracranial hemorrhage; GCS: Glasgow Coma Scale score; ISS: Injury Severity Score; No.: Number; χ2: Chi-square value; OR: Odds ratio; RR: Risk ratio; AO: Adverse outcomes. ╠Groups 1–5 proportional differences were quantified with a 2 x 5 Chi-square analysis. Groups 1–3 and ISS ≥16 proportional differences were quantified using the two-tailed Fisher exact test.

Group	No.	%	Death	p-value	χ^2^	OR	RR	AO	p-value	χ^2^	OR	RR
ICH & GCS <15	77	7.5%	9 (11.7%)	–	–	–	–	40 (52.0%)	–	–	–	–
No ICH & ISS ≥16	60	5.8%	1 (1.7%)	–	–	–	–	25 (41.7%)	–	–	–	–
ICH & GCS 15 & ISS ≥16	112	10.9%	6 (5.4%)	–	–	–	–	43 (38.4%)	–	–	–	–
No ICH & ISS <16	629	61.0%	11 (1.8%)	–	–	–	–	123 (19.6%)	–	–	–	–
ICH & GCS 15 & ISS <16	153	14.8%	1 (0.7%)	–	–	–	–	27 (17.7%)	–	–	–	–
Total	1,031	100	28 (2.7%)	<0.0001	31.4^╠^	–	–	258 (25.0%)	<0.0001	63.7^╠^	–	–
Groups 1–3	249	24.2%	16 (6.4%)	–	–	–	–	108 (43.4%)	–	–	–	–
Groups 4 & 5	782	75.8%	12 (1.5%)	<0.0001	17.1	4.4	4.2	150 (19.2%)	<0.0001	58.9	3.2	2.3
ISS ≥16	218	21.1%	15 (6.9%)	–	–	–	–	101 (46.3%)	–	–	–	–
ISS <16	813	78.9%	13 (1.6%)	<0.0001	18.2	4.6	4.3	157 (19.3%)	<0.0001	66.9	3.6	2.4

EMR audits for the scene and hospital transfer consultation patients in group 2) No ICH & ISS ≥16 and group 3) ICH & GCS 15 & ISS ≥16 are displayed in Table 7. The total audits required to assess undertriage were 129 (scene and transfer patient with No ICH & ISS ≥16 and transfer patient with ICH & GCS 15 & ISS ≥16). Of the 60 patients with No ICH & ISS ≥16, 46 (76.7%) had normal vital signs and neurologic function (39 with rib fractures and normal breath sounds and seven with face or spine fractures).

**Table 7:** Trauma consultation patient ICH-GCS-ISS triage categorical electronic medical record audit results Scene indicates that the trauma patient was transported from the site where the injury occurred directly to the trauma center's emergency department. ICH: Intracranial hemorrhage; GCS: Glasgow Coma Scale score; ISS: Injury Severity Score; ICU: Intensive care unit.

Group	Triage Assignment
Scene	–
No ICH & ISS ≥16	6/23 had undertriage (4 had extremity paralysis and 2 required an emergency splenectomy)
ICH & GCS 15 & ISS ≥16	12/43 required ICU admission owing to intracranial mass effect; triage was appropriate because the admission GCS was 15
Hospital transfer	–
No ICH & ISS ≥16	8/37 had undertriage (2 had rib fractures and tachypnea, 4 had extremity paralysis, and 2 required an emergency splenectomy)
ICH & GCS 15 & ISS ≥16	22/69 required ICU admission owing to intracranial mass effect; this would have been known at the referring hospital, so these patients represent undertriage

The undertriage counts for all consultation patients are depicted in Table 8.

**Table 8:** Trauma consult patient ICH-GCS-ISS categorical undertriage computation ICH: Intracranial hemorrhage; GCS: Glasgow Coma Scale score; ISS: Injury Severity Score; ICU: Intensive care unit.

Categories	Total No.	Undertriage
Scene undertriage	–	–
ICH & GCS <15	31	31
No ICH & ISS ≥16	23	6
ICH & GCS 15 & ISS ≥16	43	0
No ICH & ISS <16	246	0
ICH & GCS 15 & ISS <16	58	0
Total	401	37 (9.2%)
Transfer undertriage	–	–
ICH & GCS <15	46	46
No ICH & ISS ≥16	37	8
ICH & GCS 15 & ISS ≥16	69	22
No ICH & ISS <16	383	0
ICH & GCS 15 & ISS <16	95	0
Total	630	76 (12.1%)
Scene+transfer undertriage	–	–
Scene: ICH & GCS <15	31	31
Scene: No ICH & ISS ≥16	23	6
Scene: ICH & GCS 15 & ISS ≥16	43	0
Scene: No ICH & ISS <16	246	0
Scene: ICH & GCS 15 & ISS <16	58	0
Transfer: ICH & GCS <15	46	46
Transfer: No ICH & ISS ≥16	37	8
Transfer: ICH & GCS 15 & ISS ≥16	69	22
Transfer: No ICH & ISS <16	383	0
Transfer: ICH & GCS 15 & ISS <16	95	0
Total	1031	113 (11.0%)

Using the ICH-GCS-ISS method, the undertriage proportion was 11.0% (113/1,031). The modified Cribari consultation patient ISS ≥16 methods had a higher undertriage proportion (21.1%) than the consultation patient ICH-GCS-ISS categorization method (11.0%; p<0.0001; OR=2.2; RR=1.9).

Of the trauma consultation patients, ICH was found in 33.2% of patients (342/1,031) and 46.2% (158/342) with ICH had an ISS ≥16. A comparison of ISS ≥16 consultation patients without and with ICH is presented in Table 9. When compared with ISS ≥16 consultation patients without ICH, those with ISS ≥16 and ICH had higher proportions of fall mechanism, age ≥70 years, PEMC, GCS <15, ISS ≥25, discharge disposition other than home, discharge to a skilled nursing facility or long-term acute care, and discharge to a skilled nursing facility or long-term acute care or died.

**Table 9:** Comparisons of ISS ≥16 consultation patients without and with intracranial hemorrhage Adverse outcomes include hospital death, ICU stay ≥two days, or hospital stay >five days ISS: Injury severity score; ICH: Intracranial hemorrhage; χ2: Chi-square value generated using the two-tailed Fisher exact test; OR: Odds ratio; RR: Risk ratio; PEMC: Preexisting medical condition; GCS: Glasgow Coma Scale score; SNF: Skilled nursing facility; LTAC: Long-term acute care; DC: Discharge.

Variable	ICH – No	ICH – Yes	p-value	χ^2^	OR	RR
Number	60 (27.5%)	158 (72.5%)	–	–	–	–
Fall mechanism	43 (71.7%)	149 (94.3%)	<0.0001	21.2	6.5	1.3
Age ≥70 years	23 (38.3%)	114 (72.2%)	<0.0001	21.3	4.2	1.9
Any PEMC	25 (41.7%)	99 (62.7%)	0.0052	7.8	2.4	1.5
GCS <15	4 (6.7%)	46 (29.1%)	0.0004	12.4	5.8	4.4
ISS ≥25	5 (8.3%)	43 (27.2%)	0.0027	9.0	4.1	3.3
Non-home disposition	21 (35.0%)	99 (62.7%)	0.0002	13.4	3.1	1.8
SNF or LTAC DC	10 (16.7%)	71 (44.9%)	0.0001	14.9	4.1	2.7
Death, SNF, or LTAC	11 (18.3%)	82 (51.9%)	<0.0001	20.0	4.8	2.8

A summary of the various ISS ≥16 undertriage methods computed in the current study and their results are shown in Table 10.

**Table 10:** Comparison of the various ISS ≥16 methods used for computing undertriage proportions ISS: Injury severity score; UT: Undertriage; FTTA: Full trauma team activation patients; PTTA: Partial trauma team activation patients; AO: Adverse outcomes; ICH: Intracranial hemorrhage; GCS: Glasgow Coma Scale score.

ISS ≥16 Undertriage Method	Numerator Count	Denominator Count	UT %
Peng [12]	ISS ≥16 without FTTA (consultations + PTTA)	All ISS ≥16	64.2%
Modified Peng	ISS ≥16 consultations	All ISS ≥16	38.6%
Cribari [12]	ISS ≥16 with PTTA or consultation	All PTTA + consultations	21.7%
Modified Cribari	ISS ≥16 consultations	All consultations	21.1%
Modified Cribari with AO	ISS ≥16-AO consultations	All consultations	9.8%
Modified Cribari with death	ISS ≥16-death consultations	All consultations	1.5%
ICH-GCS-ISS-categorizations	Total UT count	All consultations	11.0%

## Discussion

Activation and consultation ISS ≥16 undertriage methods

The Peng method for describing trauma center undertriage was computed as the number of patients with ISS ≥16 but without full trauma team activation (partial activation plus consultation patients) divided by the total number of patients with ISS ≥16 (full trauma team activation, plus partial trauma team activation, plus consultation) [12]. The Peng undertriage computation was more than 60% which is similar to other investigations [4-7,9-11]. Of concern are the high levels and wide range of undertriage proportions in these seven studies [4-7,9-11]. The current study displayed evidence that consultation patients with ISS ≥16 are substantially different from non-consultation patients with ISS ≥16. Those group comparisons included 1) patients with ISS ≥16 undergoing full trauma team activation and patients with ISS ≥16 receiving partial trauma team activation or consultation; 2) patients with ISS ≥16 undergoing any full or partial activation and patients with ISS ≥16 receiving consultation; and 3) patients with ISS ≥16 undergoing full trauma team activation and patients with ISS ≥16 receiving consultation. This evidence implies that patients with ISS ≥16 receiving consultation are considerably different from those with ISS ≥16 undergoing trauma activation. We believe that this evidence indicates that the Peng method is highly debatable.

Numerous investigators have questioned ISS ≥16 as a reliable metric for indicating major injury and have complemented or replaced ISS ≥16 by determining those who had a need for trauma intervention [4,5,13-17]. We modified the Peng method by computing the undertriage proportion as the number of consultation patients with ISS ≥16 divided by the total number of patients with ISS ≥16 (activation plus consultation patients). This procedure has precedence in a peer-reviewed publication [8]. The modified Peng undertriage proportion in the current study represented a 40% reduction compared with the Peng undertriage proportion. This large reduction provides further evidence that the various methods for computing undertriage can have a substantial impact on the observed proportion.

Distinct from the Peng undertriage method is the Cribari undertriage method [12]. The Cribari method excludes full trauma team activation patients with ISS ≥16 and computes undertriage as the number of patients with ISS ≥16 undergoing partial trauma team activation or consultation divided by the total group of patients who had partial trauma team activation or consultation [12]. The fact that the undertriage proportion using the Cribari method in the current study is similar to that computed from data in another study (14.3%) suggests that the current study data are representative of other trauma center ISS data [5]. We have provided evidence in the current study that patients with ISS ≥16 undergoing partial trauma team activation were substantially different from those with ISS ≥16 receiving consultation. The differences included risk conditions (non-fall mechanism, age <70 years, and GCS <15) and hospital outcomes (adverse outcomes, ICU admission, and total hospital stay).

The current study has shown evidence that patients with ISS ≥16 undergoing consultation have considerably different risk conditions compared with patients with ISS ≥16 who received full or partial trauma team activation. Further, the same relative findings applied to outcomes. These observations indicate that an ISS ≥16 in consultation patients does not have the same major injury implications as this metric has for full or partial trauma team activation patients. Hence, an ISS ≥16 in consultation patients should not be commingled with an ISS ≥16 in full or partial trauma activation patients to assess undertriage. These observations have parallel support in a study by Morris et al., which showed that the need for trauma intervention in full trauma team activation patients was five times the frequency (OR, 13.0) when compared to the combined cohort of partial trauma team activation and consultation patients [5]. The same investigation demonstrated that ISS ≥15 in full trauma team activation patients was four times the frequency (OR, 7.5) when compared with the combined cohort of partial trauma team activation and consultation patients [5].

Consultation patient ISS ≥16 undertriage methods

We computed a modified Cribari undertriage method, which was the number of patients with ISS ≥16 undergoing consultation divided by the total group of patients who had a consultation. Other trauma consultation studies have provided data using the modified Cribari undertriage method, with a range of 9.1-17.2% [5,15-17]. We qualified the patients with ISS ≥16 undergoing consultation by subtending those with adverse outcomes or death. Accordingly, the ISS ≥16 with adverse outcomes and ISS ≥16 with death undertriage proportions in consultation patients were substantially lower than the undertriage proportion when using the nonqualified ISS ≥16 as the metric for undertriage. An analogous procedure has been performed in consultation with patient undertriage computations by qualifying ISS ≥16 with those who had a need for trauma intervention [15-17]. Similar to the current study, ISS ≥16 qualifications with a need for trauma intervention led to substantial reductions in the undertriage proportions when compared with the nonqualified ISS ≥16 computations (63.7-86.3% reduction) [15-17]. The consultation ISS ≥16 with death undertriage proportion could be computed for two of these studies and was <2.0%, which is the same as that in the current investigation [16,17].

It is of interest that age ≥70 years or PEMC was present in 80% of consultation patients with ISS ≥16 and an adverse outcome and in 90% of consultation patients with ISS ≥16 and death. These preinjury risk conditions generate a question as to whether these patients would have had a better outcome if they had undergone a trauma activation process.

Consultation patient ICH-GCS-ISS categorization undertriage method

The consultation patients were classified according to five mutually exclusive categories based on ICH, GCS, and ISS characteristics. The five categories were 1) ICH & GCS<15; 2) No ICH & ISS ≥16; 3) ICH & GCS 15 & ISS ≥16; 4) No ICH & ISS <16; and 5) ICH & GCS 15 & ISS <16.

Groups 1-3 had substantially greater death and adverse outcome proportions when compared to groups 4 and 5. The ISS ≥16 proportion for groups 1-3 was 90%, and the ISS <16 for groups 4 and 5 was 100%. Because the ISS was <16 for groups 4 and 5, the trauma consultation patients in the groups were considered to be appropriately triaged. Because trauma consultation patients in group 1 (GCS <15 and ICH) had a GCS violation of the institutional triage protocol, these patients indicated undertriage.

Other trauma researchers have demonstrated that most trauma consultation deaths had a decreased GCS [17] and that mortality was associated with a decreased GCS in patients with undertriage [9]. Trauma consultation patients in group 2 (No ICH & ISS ≥16) and group 3 (ICH & GCS 15 & ISS ≥16) underwent EMR audits to assess the clinical circumstances and make a determination of undertriage or appropriate triage. Of the patients in group 2 (No ICH & ISS ≥16), three-quarters had no major injury based on the EMR audits. This observation points to the imprecision of ISS ≥16 in trauma consultation patients to signify the presence of a major injury. This is consistent with the procedure where other investigators have qualified ISS ≥16 according to those who had a need for trauma intervention to render a more accurate assessment of undertriage in trauma consultation patients [15-17]. These investigations show that most trauma consultation patients with ISS ≥16 had no need for trauma intervention [15-17].

Although EMR audits showed that one-quarter of scene patients in group 3 (ICH & GCS 15 & ISS ≥16) required ICU admission owing to intracranial mass effect, these patients were considered appropriately triaged on the basis of the GCS 15 value. EMR audits showed that one-third of transfer patients in group 3 (ICH & GCS 15 & ISS ≥16) required ICU admission owing to the intracranial mass effect. These patients were considered as undertriage because the referring medical staff would have known about the presence of an intracranial mass effect.

Of note, one-third of consultation patients in the current study had ICH and 50% of those with ICH had an ISS ≥16. When contrasting ISS ≥16 consultation patients without ICH to those with ISS ≥16 and ICH, our investigation showed that those with ICH had several higher risk conditions (age ≥70 years, PEMC, GCS <15, and ISS ≥25) and were more likely to incur a discharge disposition other than home. This observation is particularly important in that patients with ICH had an increased proportion of deaths or need for discharge to a facility that could provide skilled nursing care or long-term acute care. If a trauma consultation patient undertriage investigation has no ICH designation option, those with ISS ≥16 and ICH would have commingled with other patients who had ISS ≥16 and chest, abdominal, extremity, or spine injuries. We believe that there are too many clinical and risk distinctions to combine all patients with ISS ≥16 into a single group. Of relevance, other trauma investigators have shown that undertriage patients commonly have ICH or high head/neck Abbreviated Injury Scale scores [6,10,17,43].

Interestingly, the consultation patient ICH-GCS-ISS categorical undertriage proportion is virtually identical to the consultation patient ISS ≥16 with adverse outcome undertriage proportion (one in 10). The ISS ≥16 with adverse outcome undertriage method can be employed from trauma registry data, whereas the ICH-GCS-ISS categorization undertriage method requires an audit for 10% of the patients. The ISS ≥16 with the need for trauma intervention would require an audit of all consultation patients with ISS ≥16.

ISS ≥16 audit options

ISS ≥16 is an indicator of major anatomic injuries and portends a subset of trauma consultation patients at risk for death or increased length of stay and, therefore, potential undertriage [40]. However, increased age, PEMC, GCS <15, and ICH also have associations with death or increased length of stay [40]. It is elucidating to consider that 60% of trauma consultation patients who have an AO (die or have an increased length of stay) have an ISS <16 and that over 90% of consultation patients with ISS ≥16 survive [40]. Audits from the current study showed that three-quarters of trauma consultation patients with ISS ≥16 and No ICH had physiological stability and were neurologically intact. We believe that any reliable trauma consultation patient undertriage assessment will require individual audits in targeted patient cohorts. Audits should include the initial patient injury status and the likelihood that outcomes could have been improved with trauma activation.

The audit options are considerable and the proportion of the consultation cohort requiring an audit vary from highest to lowest, as follows: 1) all ISS ≥16 patients (21%); 2) ICH-GCS-ICH categorization (13%); 3) ISS ≥16 patients with adverse outcomes (10%); 4) ISS ≥16 patients with adverse outcomes and age <70 years and no PEMC (2%); and 5) ISS ≥16 patients with death (2%). Importantly, the method of ISS ≥16 patients with a need for trauma intervention would also necessitate an audit of all trauma consultation patients with an ISS ≥16. The pros and cons of each method will need to be carefully considered by the trauma systems committee members. We believe that an important objective in trauma consultation patient undertriage is to identify systematic deficiencies in the trauma triage process. Such an intention will mandate individual patient audit assessments to secure reasonable clinical conclusions.

Limitations of the study

The principal limitation of the current study is its retrospective design. Its findings need to be compared with data that are prospectively collected and support a specific set of hypotheses. The PEMC score does not precisely reflect the values given in most publications on the Charlson Comorbidity Index. Another limitation is that the ISS was the only anatomic injury descriptor used, although other anatomic injury assessments exist. Another consideration is that the data used in the current analysis included the coronavirus disease pandemic period and did not include the entire calendar year.

## Conclusions

Consultation patients with ISS ≥16 have distinctly different risk and adverse outcome proportions compared with patients with ISS ≥16 who undergo full or partial trauma team activation. Thus, any method that assesses consultation patient undertriage using ISS ≥16 as a benchmark for major injury should not be commingled with ISS ≥16 patients who underwent full or partial trauma team activation. It is reasonable to use ISS ≥16 as an overall filter for assessing undertriage among trauma consultation patients. However, we recommend that ISS ≥16-qualified cohorts be identified to undergo individual patient audits. Considerations for trauma consultation patient auditing are those with ISS ≥16 and 1) death, 2) adverse outcomes (death or increased length of stay), 3) adverse outcomes with age <70 years and no PEMC, 4) ICH, and/or 5) GCS <15. Following the audit finding, a reasonably precise proportion of undertriage can be determined. If systematic undertriage issues are found, either the triage protocol needs to be modified or educational interactions need to take place with the emergency department personnel who assign the prehospital triage status.
